# Global disparities in pediatric neurosurgery research: a 20-year bibliometric analysis by country income group

**DOI:** 10.1007/s00381-025-06975-2

**Published:** 2025-10-10

**Authors:** Khushi H. Shah, Victor M. Lu, Toba N. Niazi

**Affiliations:** 1 Department of Neurological Surgery, University of Miami Miller School of Medicine, Miami, FL U.S.A.; 2 Department of Neurological Surgery, Nicklaus Children’s Health System, Miami, FL U.S.A.

**Keywords:** Bibliometrics, pediatric neurosurgery, high-Income country, upper-middle-income country, lower-middle-income country, low-income country

## Abstract

**Purpose:**

Pediatric neurosurgical diseases are most prevalent in low-income (LICs) and low-middle-income countries (LMICs), yet these regions have limited representation in global neurosurgical workforce. To assess whether scholarly output reflects this burden, we conducted a 20-year bibliometric analysis of pediatric neurosurgery publications, evaluating trends by country income level.

**Methods:**

We analyzed publications on “pediatric neurosurgery” indexed in Web of Science from 2005–2024. Author affiliations were categorized by World Bank income groups: high-income (HIC), upper-middle-income (UMIC), LMIC, and LIC. Lorenz curves and Gini coefficients assessed authorship inequality, with Mann–Kendall test evaluating trends over time.

**Results:**

Of 3,014 publications from 116 countries, HICs contributed 84.18% of overall authorship. In contrast, UMIC, LMIC, and LIC contributed to 16.70%, 9.59%, and 3.05%, respectively. Multi-country collaborations accounted for 23.79% of all publications, with HIC only (48.43%) partnerships most common, followed by HIC-UMIC (17.09%), and HIC-LMIC (16.24%). No LIC only partnerships were identified. HIC authorship declined from 86.82% to 77.78% over study period (p < 0.001). In contrast, UMIC, LMIC, and LIC share rose from 11.50% to 21.72%, 5.43% to 13.20%, 0.90% to 3.68%, respectively. First authorship trends mirrored overall authorship, although LIC representation remained unchanged (p = 0.138). Gini coefficients declined, indicating reduced research inequality over time (tau =  − 0.66, p < 0.001).

**Conclusion:**

Despite growing international collaborations and a gradual shift towards more equitable authorship, contributions from LICs and LMICs, particularly in first and corresponding author roles, remain limited. Investment in research capacity, mentorship, and inclusive partnerships is critical to ensure the literature reflects the populations most affected by pediatric neurosurgical disease.

## Introduction

The global burden of common pediatric neurosurgical disease, such as hydrocephalus, traumatic brain injury, epilepsy, brain tumors, cerebrovascular, and brain infections [1–6], is substantial. The majority of these affected children live in low-income (LIC) and lower-middle-income (LMICs) countries, where access to specialized care is limited. These disparities in pediatric neurosurgical disease are further exacerbated by the unequal global distribution of pediatric neurosurgeons [7]. Of the estimated 2,297 pediatric neurosurgeons worldwide, 85.6% practice in high-income countries (HICs) or upper-middle-income countries (UMICs) [7], despite only 39.3% of the global pediatric population aged 0–14 years residing in these regions [8]. In contrast, approximately 330 pediatric neurosurgeons serve a combined child population of 1.2 billion across LMICs and LICs [7].

Disparities in pediatric neurosurgical care are also likely to impact the origin of pediatric neurosurgical literature. Bibliometric analyses provide a valuable method for evaluating research output, identifying gaps in representation, and tracking publication trends over time [9]. In adult neurosurgery, prior bibliometric studies have identified imbalances in authorship by country income level, particularly affecting researchers from LICs and LMICs [10–12]. Within pediatric neurosurgery, however, bibliometric analyses remain limited. To date, only one study has examined global authorship trends, focusing exclusively on pediatric spine surgery without stratifying data by country income level [13]. Although qualitative reviews and workforce surveys have described regional variations and inequities in access to pediatric neurosurgical care [7, 14–16], no comprehensive bibliometric study has evaluated global authorship patterns across the broader landscape of pediatric neurosurgical research.

In this study, we present a 20-year bibliometric analysis of pediatric neurosurgery publications indexed in the Web of Science from 2005 to 2024. We examine temporal trends in authorship by country income level to quantify disparities in scholarly representation and inform future strategies aimed at advancing equity in global pediatric neurosurgical publishing.

## Methods

### Inclusion and exclusion criteria

This bibliometric review was performed according to the BIBLIO guidelines. A search query was performed on Web of Science using the search term "pediatric neurosurgery”. Results were limited to those published between January 1, 2005, and December 31, 2024. All publication types and languages were included. Ten articles were excluded because although they were identified in initial search, we noted that they were indexed in 2025, outside the study time range. Institutional Review Board approval was not required for this study, as all data were obtained from publicly available sources. Patient consent was not applicable as no patient data or protected health information was obtained.

### Data extraction

Pertinent data such as article title, year of publication, journal, open-access status, publication type, language, author affiliations, and article keywords were extracted from Web of Science. Authorship details such as overall authorship, first authorship, and corresponding authorship were extracted. Corresponding authorship was defined as the last author in author list. Country names were extracted from author affiliations for first, corresponding, and all contributing authors. Multi-country partnerships were defined as author contributions from more than one country. Author countries were stratified using 2024–2025 World Bank income classifications into the following income categories: high-income countries (HIC), upper-middle-income countries (UMIC), lower-middle-income countries (LMIC), and low-income countries (LIC)[17]. Annual population data on countries was obtained through the World Bank for 2005–2023. Population data from 2024 was unavailable at the time of analysis. This was used to compare the cumulative share of research output to the cumulative global population by country income groups [17].

### Statistical analysis

Categorical variables were summarized using frequencies and percentages and compared using Chi-square test. For comparisons across multiple groups with small sample sizes or low expected cell counts, Fisher’s exact test with Monte Carlo simulation (10,000 replicates, seed = 5) was performed to assess differences in proportions. Continuous variables were analyzed for normality and were compared using either ANOVA or Kruskal Wallis tests as applicable. VOS-viewer was used to visualize co-occurrence of keywords and map collaborations between countries [18]. A minimum threshold of 5 occurrences was set for this mapping. Authorship inequality was visualized using Lorenz curves plotting the cumulative share of research output against the share of global population by country income groups [19]. Gini coefficients were calculated for each year studied to summarize the extent of global authorship inequality, with Mann–Kendall trend test to assess trends in Gini coefficients over time [19]. Statistical significance was set at a p value < 0.05. Statistical analyses were performed using Python version 3.11.5 (MacOS) and GraphPad Prism.

## Results

### Overall bibliometric characteristics

A total of 3014 publications met inclusion criteria, of which 30.82% were published as open access. Publication volume increased from 2005 to 2007, declined through the mid-2010s, and then steadily rose from 2018, peaking in 2024 (9.22%). Most publications were original articles (79.53%), followed by reviews (13.21%), and editorials (3.25%). English was the predominant language (98.54%), followed by French (0.60%) with all other languages contributing less than 0.5% each (Table 1).

**Table 1:** Descriptive summary of included publications

Variables	Value
Total publications	3014
Open access	929 (30.82%)
Year of publication	
2005	183 (6.07%)
2006	197 (6.54%)
2007	222 (7.37%)
2008	103 (3.42%)
2009	69 (2.29%)
2010	90 (2.99%)
2011	93 (3.09%)
2012	87 (2.89%)
2013	94 (3.12%)
2014	108 (3.58%)
2015	86 (2.85%)
2016	105 (3.48%)
2017	98 (3.25%)
2018	151 (5.01%)
2019	131 (4.35%)
2020	197 (6.54%)
2021	214 (7.10%)
2022	237 (7.86%)
2023	271 (8.99%)
2024	278 (9.22%)
Study type	
Article	2397 (79.53%)
Review	398 (13.21%)
Editorial material	98 (3.25%)
Meeting abstract	38 (1.26%)
Letter	33 (1.09%)
Proceedings papers	19 (0.63%)
Book review	8 (0.27%)
Meeting	7 (0.23%)
Biographical items	6 (0.20%)
Correction	4 (0.13%)
Reprint	4 (0.13%)
News item	2 (0.07%)
Study language	
English	2970 (98.54%)
French	18 (0.60%)
Spanish	15 (0.50%)
German	7 (0.23%)
Turkish	4 (0.13%)
Hungarian	3 (0.10%)
Portuguese	3 (0.10%)
Japanese	1 (0.03%)
Czech	1 (0.03%)
Italian	1 (0.03%)
Korean	1 (0.03%)

### Keyword analysis

**Fig. 1:**
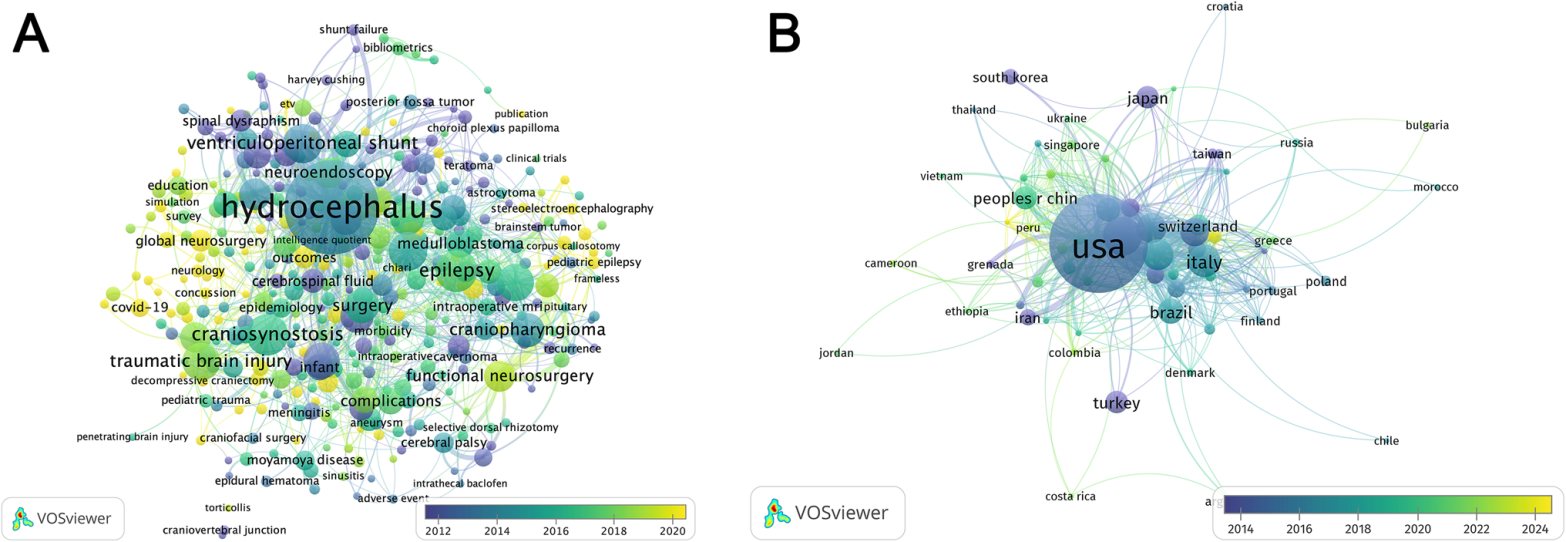
Mapping of co-occurrences of (**A**) author keywords and (**B**) countries of author affiliation. Visualization was performed using VOSviewer with a minimum occurrence threshold of n = 5

A total of 5519 keywords were identified across 3014 publications. For subgroup analysis, we restricted the dataset to keywords occurring at least 5 times, yielding 375 unique terms. Nine general terms – “pediatric neurosurgery”, “paedriatric neurosurgery”, “pediatric surgery”, “neurosurgery”, “pediatric”, “pediatrics”, “children”, “child”, “childhood” – were excluded from thematic analysis due to their broad nature, resulting in 366 keywords used for mapping with a total of 4815 occurrences (Fig. 1A). The most frequent keywords were hydrocephalus (74.25%), craniosynostosis (19.51%), epilepsy (19.51%), ventriculoperitoneal shunt (17.62%), and brain tumor (17.34%). All keywords were categorized under the following topics: tumor (14.35%), CSF disorders and neuroendoscopy (14.31%), surgical techniques (11.73%), congenital malformations (11.30%), outcomes (6.92%), neurotrauma and critical care (6.65%), epilepsy and functional neurosurgery (5.86%), vascular (4.13%), global neurosurgery (3.68%), infection (2.66%), spine (2.55%), education (1.37%), and other (14.50%) (Table 2).

**Table 2:** Categorization of keywords into thematic categories

Countries	Number of keywords	Number of occurrences
Congenital malformations	35	544 (11.30%)
CSF disorders and neuroendoscopy	27	689 (14.31%)
Education	6	66 (1.37%)
Epilepsy and functional neurosurgery	12	282 (5.86%)
Global neurosurgery and health systems	18	177 (3.68%)
Infection	10	128 (2.66%)
Neurotrauma and critical care	23	320 (6.65%)
Other	81	698 (14.50%)
Patient outcomes	21	333 (6.92%)
Spine (other than congenital)	12	123 (2.55%)
Surgical techniques	60	565 (11.73%)
Tumor	44	691 (14.35%)
Vascular	17	199 (4.13%)
Total	366	4815

### Authorship characteristics by country income level

Country information was available for 2952 publications. Authorship spanned 116 countries. Most publications involved contributors from a single country (76.22%). Most publications had authors affiliated with HICs (84.18%), followed by UMICs (16.70%), LMICs (9.59%), and LICs (3.05%). Among HICs, the highest contributions came from the United States (49.90%), Canada (7.89%), and United Kingdom (5.86%). Within UMICs, Türkiye (3.83%), Brazil (3.62%), and China (3.05%) had the greatest representation. India (4.51%), Egypt (0.91%), and Nigeria (0.75%) were the top contributors from LMICs, while Niger (0.75%), Chad (0.71%), and Mali (0.54%) accounted for the most publications among LICs (Table 3). Mapping of countries of author affiliations is provided in Fig. 1B.

**Table 3:** Countries with authorship contribution greater than 0.5% (data available for n = 2952)

Countries	Income classification	Value
United States	High income	1473 (49.90%)
Canada	High income	233 (7.89%)
United Kingdom	High income	173 (5.86%)
Italy	High income	167 (5.66%)
France	High income	167 (5.66%)
Germany	High income	154 (5.22%)
India	Lower middle income	133 (4.51%)
Türkiye	Upper middle income	113 (3.83%)
Brazil	Upper middle income	107 (3.62%)
China	Upper middle income	90 (3.05%)
Japan	High income	83 (2.81%)
Switzerland	High income	71 (2.41%)
Iran	Upper middle income	65 (2.20%)
Israel	High income	63 (2.13%)
Australia	High income	58 (1.96%)
Netherlands	High income	52 (1.76%)
Spain	High income	50 (1.69%)
South Korea	High income	40 (1.36%)
Oman	High income	32 (1.08%)
Egypt	Lower middle income	27 (0.91%)
Sweden	High income	22 (0.75%)
Austria	High income	22 (0.75%)
Niger	Low income	22 (0.75%)
Nigeria	Lower middle income	22 (0.75%)
Mexico	Upper middle income	21 (0.71%)
Norway	High income	21 (0.71%)
Chad	Low income	21 (0.71%)
Saudi Arabia	High income	20 (0.68%)
Poland	High income	19 (0.64%)
Taiwan	High income	18 (0.61%)
Georgia	Upper middle income	17 (0.58%)
Pakistan	Lower middle income	16 (0.54%)
Mali	Low income	16 (0.54%)
Jordan	Lower middle income	15 (0.51%)
Uganda	Low income	15 (0.51%)
South Africa	Upper middle income	15 (0.51%)

Among multi-country collaborations (n = 702, 23.78%), HIC involvement was present in 92.31% of publications (Table 4). The most frequent partnerships were between HICs alone (48.3%), followed by collaborations between HICs and UMICs (17.09%) and between HICs and LMICs (16.24%). Among studies from LICs, the most common collaborations were with HICs (4.42%), followed by UMICs (1.99%) and LMICs (1.85%) with no studies involving LIC-only collaborations. Four-income-level collaborations involving HIC, UMIC, LMIC, and LIC authors only occurred in 1.28% (n = 9) of all multi-country publications.

Among first authors, 84.18% were affiliated with HICs, followed by UMICs (10.70%), LMICs (3.96%), and LICs (1.15%). Corresponding authors showed a similar distribution, with 91.62% from HICs, 13.67% from UMICs, 6.31% from LMICs, and 0.89% from LICs. Most publications (95.53%) had a single corresponding country, while 4.40% involved 2 countries, and 0.07% involved 3 countries.

**Table 4:** Authorship distribution by country income group per World Bank classification

Variables	Value
**Overall authorship:**	
n	2952
Number of countries involved	
1	2250 (76.22%)
2	515 (17.45%)
3	118 (4.00%)
4 or more	69 (2.34%)
Income level involvement	
HIC	2485 (84.18%)
UMIC	493 (16.70%)
LMIC	283 (9.59%)
LIC	90 (3.05%)
Multi-country partnerships	702 (23.79%)
HIC involvement	648 (92.31%)
UMIC involvement	189 (26.92%)
LMIC involvement	178 (25.36%)
LIC involvement	84 (11.97%)
Multi-country partnership details	
HIC only	340 (48.43%)
HIC-UMIC	120 (17.09%)
HIC-LMIC	114 (16.24%)
HIC-LIC	31 (4.42%)
UMIC only	13 (1.85%)
UMIC-LMIC	9 (1.28%)
UMIC-LIC	14 (1.99%)
LMIC only	3 (0.43%)
LMIC-LIC	13 (1.85%)
LIC only	0 (0.00%)
3 country income levels	35 (4.99%)
LIC-LMIC-UMIC-HIC	9 (1.28%)
**First authorship:**	
n	2952
Income level involvement	
HIC	2485 (84.18%)
UMIC	316 (10.70%)
LMIC	117 (3.96%)
LIC	34 (1.15%)
**Corresponding authorship:**	
n	2933
Number of countries involved	
1	2802 (95.53%)
2	129 (4.40%)
3	2 (0.07%)
Income level involvement	
HIC	2394 (81.62%)
UMIC	401 (13.67%)
LMIC	185 (6.31%)
LIC	26 (0.89%)

#### Temporal trends in pediatric neurosurgical literature

The number of pediatric neurosurgery publications increased across each 5-year time interval from 2005 to 2024, rising from 774 in 2004–2009 to 472 in 2010–2014, 571 in 2015–2019 and 1,197 in 2020–2024 (p < 0.001). Multi-country income level partnerships increased significantly from 15.95% in 2004–2009 to 45.44% in 2020–2024 (p < 0.001). During the same period, the proportion of overall authorship from HICs declined from 86.82% in 2005–2009 to 77.78% in 2020–2024 (p < 0.001). In contrast, contributions from UMIC, LMIC, and LIC increased, rising from 11.50% to 21.72% for UMICs, 5.43% to 13.20% for LMICs, and 0.90% to 3.68% for LICs between 2005–2009 and 2020–2024, respectively (all p < 0.001) (Table 5).

**Table 5:** Temporal trends in publication characteristics

Variables	2004–2009	2010–2014	2015–2019	2020–2024	P value
Number of publications	774	472	571	1197	** < 0.001**
**Overall authorship**					
Income level involvement					
HIC	672 (86.82%)	405 (85.81%)	477 (83.54%)	931 (77.78%)	** < 0.001**
UMIC	89 (11.50%)	59 (12.50%)	85 (14.89%)	260 (21.72%)	** < 0.001**
LMIC	42 (5.43%)	40 (8.47%)	43 (7.53%)	158 (13.20%)	** < 0.001**
LIC	7 (0.90%)	17 (3.60%)	22 (3.85%)	44 (3.68%)	**0.001**
Number of countries in authorship per publication, mean, SD	1.17 ± 0.45	1.32 ± 0.61	1.42 ± 0.97	1.49 ± 1.26	** < 0.001**
Multi-country income level partnerships	112 (15.95%)	120 (17.09%)	151 (21.51%)	319 (45.44%)	** < 0.001**
**First authorship**					
Income level involvement					** < 0.001**
HIC	672 (86.82%)	405 (85.81%)	477 (83.54%)	931 (77.78%)	** < 0.001**
UMIC	62 (8.01%)	23 (4.87%)	59 (10.33%)	172 (14.37%)	** < 0.001**
LMIC	24 (3.10%)	21 (4.45%)	10 (1.75%)	62 (5.18%)	**0.003**
LIC	4 (0.52%)	9 (1.91%)	7 (1.23%)	14 (1.17%)	0.138
**Corresponding authorship**					
Income level involvement					
HIC	668 (86.30%)	386 (81.78%)	459 (80.39%)	881 (73.60%)	** < 0.001**
UMIC	71 (9.17%)	48 (10.17%)	71 (12.43%)	211 (17.63%)	** < 0.001**
LMIC	35 (4.52%)	30 (6.36%)	26 (4.55%)	94 (7.85%)	**0.007**
LIC	3 (0.39%)	0 (0.00%)	7 (1.23%)	16 (1.34%)	**0.009**

Bold entries signify statistical significance, p < 0.05

HIC, high-income country; UMIC, upper-middle-income country; LMIC, lower-middle-income country; LIC, low-income country

A similar trend was observed in first authorship, with representation from HICs decreasing from 86.82% in 2004–2009 to 77.78% in 2020–2024 (p < 0.001). Contributions from UMICs and LMICs increased from 8.01% to 14.37% (p < 0.001) and 3.01% to 5.18% (p = 0.002) within the same time period. However, there was no statistically significant change in proportion of first authors from LICs over time, with values remaining low at 0.52% in 2004–2009, 1.91% in 2010–2014, 1.23% in 2015–2019, and 1.17% in 2020–2024 (p = 0.138).

With regards to corresponding authorship, representation from HICs decreased from 86.30% in 2004–2009 to 73.60% in 2020–2024 (p < 0.001), while contributions from UMICs (9.17% to 17.63%, p < 0.001), LMICs (4.52% to 7.85%, p = 0.007), and LICs (0.39% to 1.34%, p = 0.009) all increased over the same period.

#### Trend in authorship inequality

**Fig. 2:**
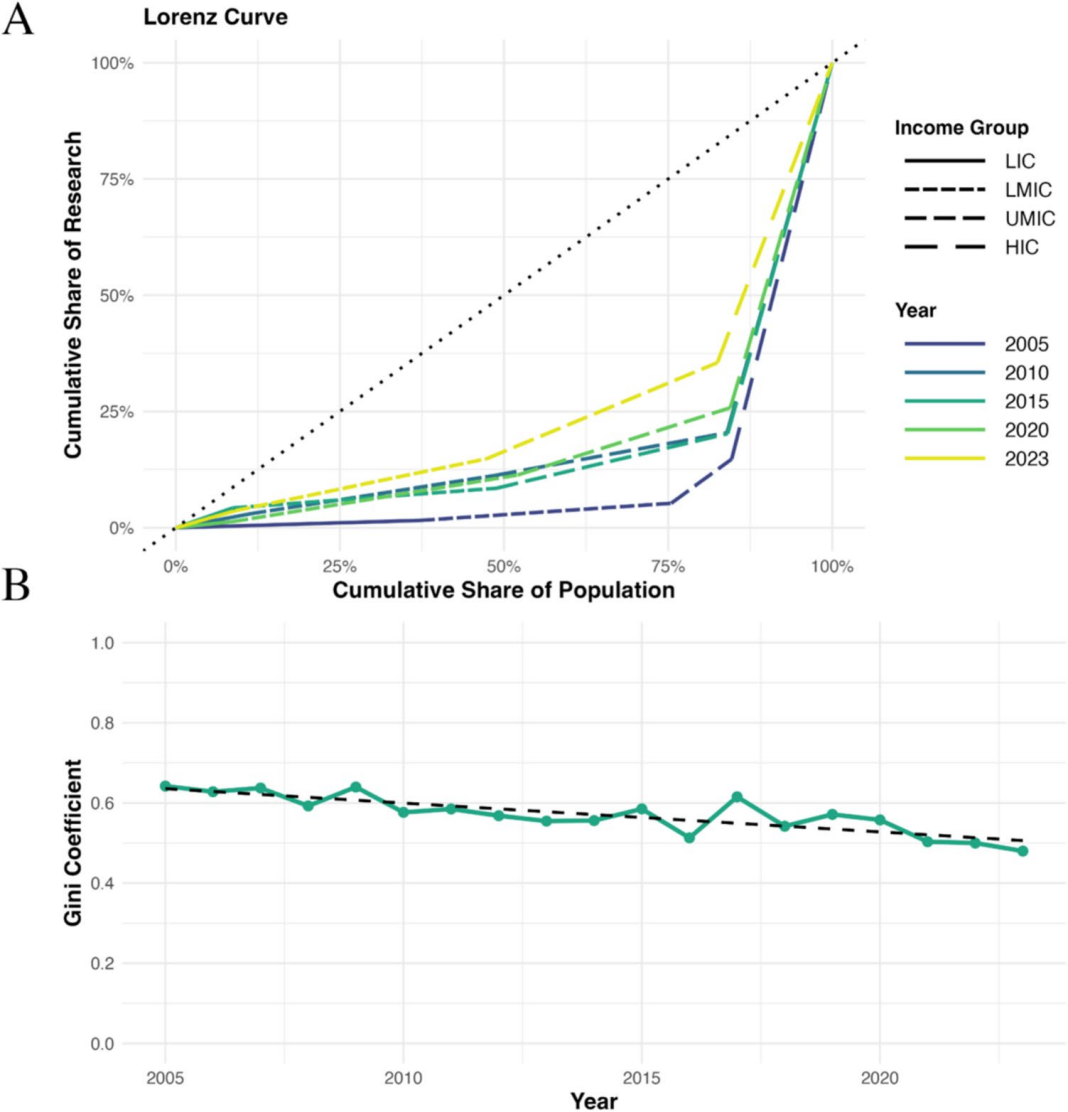
**A**) Lorenz curves and **B**) corresponding Gini coefficients illustrating disparities in pediatric neurosurgery research output relative to population share, stratified by World Bank country income groups (2005–2023)

Lorenz curves assessing authorship inequality for the years 2005, 2010, 2015, 2020, and 2023 are shown in Fig. 2A, with each curve deviating from the line of equality. Gini coefficients declined over time (Fig. 2B**)**, and Mann Kendall test demonstrated a significant trend of declining authorship inequality across the study period (τ = –0.66, z = –3.92, p < 0.001).

## Discussion

Children make up approximately one-quarter of the global population, with more than 60% residing in LMICs and LICs [8]. In these regions, as much as 85% of children will have a surgically treatable condition by the age of 15 years [20]. Yet, most child health programs fail to address injuries, congenital abnormalities, and surgical infections, which are key drivers of preventable childhood disability and death childhood [20]. Pediatric neurosurgery, in particular, remains critically under resourced with an estimated ratio of 1 neurosurgeon per 3.6 million children in LMICs and LICs [7]. This staggering workforce gap underscores the urgent need for locally generated research to inform context-specific policy, training, and care delivery. Ideally, authorship and academic representation from LICs and LMICs should reflect the global burden of pediatric neurosurgical disease they disproportionately bear. However, reliance on literature produced in HICs, where clinical resources, infrastructure, and treatment approaches differ, risks perpetuating misaligned or ineffective care models. To evaluate the extent of this mismatch, we conducted a 20-year bibliometric analysis to assess global authorship trends in pediatric neurosurgery and measure progress toward more equitable scholarly representation.

### Study overview

In this bibliometric analysis of 3,014 pediatric neurosurgical publications from 2005–2024, we found that authors from HICs were involved in over 84.14% of publications, while UMIC, LMIC, and LIC contributed to 16.70%, 9.59% and 3.05% of publications, respectively. Although overall publication volume increased, authorship remained dominated by HICs. First and corresponding authors from LICs accounted for just 1.15% and 0.89% of publications, respectively. Among collaborative publications (n = 702), 92.31% included HIC authors, whereas only 9.11% involved authors from LICs. HIC-HIC (48.43%) partnerships were most common, followed by HIC-UMIC (17.09%) and HIC-LMIC (16.24%), with no LIC only collaborations. Authorship inequality declined modestly over time, as demonstrated by improving Gini coefficients, yet disparities remain substantial. These findings reflect persistent imbalances in global pediatric neurosurgical scholarship and emphasize the need for more inclusive authorship and academic representation.

### Keywords and thematic analysis

Tumor (14.35%) and CSF disorders and neuroendoscopy (14.31%) were the most frequently represented themes in our keyword analysis, indicating that future training endeavors and capacity-building initiatives may reasonably choose to prioritize these domains first.

### Authorship by country income level

We show that overall, first, and corresponding authorship in pediatric neurosurgical publications are dominated by HICs (84.18%, 84.18%, 95.53%, respectively). In contrast, authors from LICs contributed to only 3.05% of overall authorship, 1.15% of first authorship, and just 0.89% of corresponding authorship. Our data aligns with prior studies in both adult and pediatric neurosurgery analyzing LMIC and LIC contributions to literature [11, 12]. This discrepancy in overall authorship and corresponding authorship for LICs is particularly concerning.

Beyond infrastructure and funding limitations, these disparities may also reflect epistemic injustices in global academic publishing [21]. Authors from LICs and LMICs often face credibility deficits where their expertise is undervalued unless validated through institutions in HICs [21]. HIC-shaped editorial standards and publication norms often disadvantage LIC-led research, especially when focused on region-specific issue [22]. Addressing these disparities will require not only expanded capacity-building, but also intentional efforts to deconstruct structural barriers and elevate LMIC leadership in the pediatric neurosurgical literature.

### Global collaborations

Our findings show that multi-country income level partnerships in pediatric neurosurgery have increased over the past two decades from 15.95% in 2005–2009 to 45.44% in 2020–2024. However, our data show that these collaborations remain overwhelmingly centered around HICs. Among the 702 multi-country publications, 92.31% included HIC-affiliated authors, and nearly half (48.43%) were collaborations between HICs alone. By contrast, only 9.11% of collaborative publications involved authors from LICs and no LIC only partnerships were identified.

These patterns reflect broader concerns in global academic neurosurgery, where collaborations may be international in scope but unequal in structure. Prior analyses have found that in many LMIC-HIC partnerships, local investigators are often relegated to data collection roles, while study design, analysis, and senior authorship remain HIC-led [12, 23, 24]. This dynamic not only marginalizes local expertise but may also lead to research that is less aligned with local clinical needs [24].

Despite these challenges, there is strong interest in cross-border collaboration. In a survey of surgeon members of several international neurosurgical and general pediatric societies, 89% of all respondents, and 99% of respondents from LICs and LMICs, indicated a desire for shared collaboration [7]. Moreover, in a survey of pediatric neurosurgeons on the AANS/CNS pediatrics roster, 74% expressed interest in global partnerships, but 43% reported difficulty identifying international collaborators [25]. Strengthening local neurosurgical societies, registries, and mentorship networks can help overcome these barriers. Equally important is shifting collaboration models to prioritize shared leadership, authorship parity, and early involvement of LMIC investigators in study conception and design. Major international neurosurgical societies can play a pivotal role in bridging gaps by fostering networks that connect pediatric neurosurgeons across countries and support collaborative projects aligned with the needs of under-resourced regions.

### Authorship inequality

The findings of this study align with prior neurosurgical bibliometric analyses encompassing both adult and pediatric neurosurgery demonstrating persistent disparities in global neurosurgical research output by country income level [10–12]. While our analysis revealed a modest decline in authorship inequality over the past two decades, as indicated by decreasing Gini coefficients, absolute disparities remain stark. Despite accounting for the majority of the global pediatric population and disease burden [7], authors from LICs and LMICs remain vastly underrepresented in both contributing and lead authorship roles.

Multiple structural and systemic barriers contribute to this inequality. A major factor is the lack of research infrastructure and protected academic time in many LIC and LMIC settings. Pediatric neurosurgeons in these regions often manage overwhelming clinical volumes, frequently serving as the only provider for millions of children. A 2018 survey estimated a ratio of 1 pediatric neurosurgeon per 3.6 million children across LICs and LMICs, with some countries in Africa reporting only 1 pediatric-trained neurosurgeon for more than 30 million children [7]. In-country assessments further highlight this shortage; for instance, in one LIC, a single pediatric neurosurgeon serves over 58 million children [26]. These constraints severely limit capacity for academic activity, data collection, and manuscript preparation. Investment in local infrastructure is critical to enabling scholarly participation.

In parallel, the phenomenon of “brain drain” further erodes academic capacity [27]. Emigration of trained neurosurgeons from LMICs to HICs in search of academic, training, or financial opportunities reduces the local workforce and hinders sustainable research ecosystems. A recent study from Pakistan found that 64% of neurosurgery trainees intended to emigrate, citing inadequate academic infrastructure and limited access to fellowships as key factors [28]. Retention of academic talent may be improved by establishing in-country career development pathways, expanding regional fellowship opportunities, and creating academic re-entry support for returning trainees.

Mentorship remains another critical, and often missing, element. Early-career researchers in LICs and LMICs may have limited access to experienced academic mentors, structured training in research methodology, or international scholarly networks. While global mentorship programs exist, many remain informal, short-term, or unidirectional [29, 30]. Sustainable models should emphasize long-term, bidirectional mentorship that supports shared academic output and builds local leadership [31].

Finally, barriers related to language, journal access, and publishing costs contribute to inequitable authorship representation. The predominance of English in scientific publishing, coupled with high article processing charges (APCs), often excludes non-native English speakers and researchers without institutional support. Although open-access models aim to increase visibility [32, 33], inconsistent APC waivers [34] limit their accessibility in low-resource settings. Journals can play an important role in mitigating these challenges by expanding language editing services, offering consistent fee waivers for LIC/LMIC authors, and increasing representation of LMIC investigators on editorial boards to guide inclusive publication practices.

Taken together, addressing authorship inequality will require coordinated investment in local capacity, inclusive mentorship, and structural changes to global research and publishing frameworks. Equitable academic representation ensures that the pediatric neurosurgical literature reflects not only the geography of disease, but also the priorities, experiences, and innovations of the communities most affected.

### Limitations

This study has several limitations. Although Web of Science is a widely used indexing platform, it may underrepresent pediatric neurosurgical literature from LICs and LMICs, particularly work published in regional or non-indexed journals. As a result, contributions from these settings may be underestimated. Country income classification was based on author affiliation, which may not accurately reflect the context of the research. For instance, trainees from LMICs working temporarily in HIC institutions may appear as HIC-affiliated authors. While our search included all languages indexed in Web of Science, the predominance of English-language journals may introduce linguistic bias and overlook valuable non-English contributions. Although we categorized extracted keywords into thematic groups to provide further insight, some overlap between categories may exist. Certain keywords could reasonably fit into more than one domain or, conversely, did not align neatly with any specific category and were therefore grouped under “other.” As such, while this thematic organization highlights broad research trends, some overlap of topics is possible. Finally, population-adjusted analyses were limited to World Bank data through 2023, excluding the final study year (2024) from those comparisons since population data for that year was unavailable at the time of analysis. Despite these limitations, this study provides a comprehensive, 20-year overview of global pediatric neurosurgical authorship, offering novel insight into persistent disparities in scholarly representation.

## Conclusion

Although multi-country partnerships have grown over the past two decades, authorship remains heavily dominated by HICs. While representation from UMICs, LMICs, and LICs has improved, progress has been limited, particularly in LIC first author roles and LIC only multi-country collaborations. These results underscore the need for targeted efforts that support equitable authorship, sustainable research capacity, and greater inclusion of LMIC and LIC voices in shaping the pediatric neurosurgical literature.

## Data Availability

No datasets were generated or analysed during the current study.
